# Supplementary material for: Comparative Genomic Analysis of Cold-Water Coral-Derived Sulfitobacter faviae: Insights into Their Habitat Adaptation and Metabolism
Source: Mar Drugs. 2023 May 19;21(5):309. doi: 10.3390/md21050309 (PMC10221251; doi:10.3390/md21050309)
Supplement: Supplementary file 1 [file marinedrugs-21-00309-s001.zip › marinedrugs-2391909-supplementary.pdf]

Table S1. Information of *Sulfitobacter* from genomes assembled from pure culture (PC) or metagenomic data (MAG**)**. NA: not available.

Strain	contig	CDS	Size (Mbp)	GC%	Source	Isolation	accessions	Reference
Jannaschia aquimarina GSW-M26^T	81	3,987	4.10	66.40	PC	seawater off the southern coast of Korea	GCA_000877395.1	[1]
Jannaschia formosa 12N15^T	133	4,456	4.73	69.60	PC	marine saltern sediment	GCA_003340555.1	[2]
Sulfitobacter aestuariivivens TSTF-M16^T	14	4,057	4.20	59	PC	tidal flat sediment	GCA_014763045.1	[3]
Sulfitobacter alexandrii AM1-D1^T	6	4,464	4.70	64.6	PC	Myzozoa Cavalier *Alexandrium minutum*	GCA_001886735.1	[4]
Sulfitobacter algicola 1151^T	70	3,878	3.97	51.8	pc	green algae from Weihai coastal seawater	GCA_013315265.1	[5]
Sulfitobacter brevis DSM 11443^T	68	3,977	4.24	58.4	PC	Ekho Lake water	GCA_900112755.1	[6]
Sulfitobacter delicatus DSM 16477^T	34	3,807	3.94	60.80	PC	Echinodermata *Stellaster equestris*	GCA_900102535.1	[7]
Sulfitobacter donghicola DSW-25	9	3,347	3.54	55.20	NA	coastal seawater	GCA_000622405.1	NA
Sulfitobacter dubius DSM 16472^T	21	3,520	3.67	60.20	PC	Monocotyledoneae *Zostera marina*	GCA_900113435.1	[7]
Sulfitobacter geojensis TR60-85	21	4,066	4.32	57.60	MAG	The surface seawater at the Pearl River Estuary	GCA_016814695.1	[8]
Sulfitobacter guttiformis KCTC 32187	4	3,701	3.98	56.10	NA	coastal sea water	GCA_000622425.1	NA
Sulfitobacter indolifex DSM 14862^T	8	4,211	4.50	59.20	PC	Seawater from the North Sea by Helgoland	GCF_022788665.1	NA
Sulfitobacter litoralis DSM 17584^T	63	3,510	3.68	58.20	PC	East sea water	GCA_900103185.1	[9]
Sulfitobacter marinus DSM 23422^T	12	3,417	3.57	56.90	NA	Sea water	GCA_900116285.1	NA
Sulfitobacter maritimus S0837^T	38	3,565	3.79	59.80	PC	coastal water sediment	GCA_013346665.1	[10]
Sulfitobacter mediterraneus SC7-37	6	3,873	4.05	58.48	MAG	Ochrophyta *Skeletonema costatum* from Estuary	GCA_021043225.1	[8]
Sulfitobacter sp. strain EL44	14	4,006	4.20	58.10	PC	Gorgonian coral *Eunicella labiate*	GCA_900313045.1	[11-13]
Sulfitobacter noctilucae NB-68^T	14	3,664	3.91	58.30	PC	coastal seawater around Geoje island	GCA_000622365.1	[14]

Sulfitobacter noctilucicola NB-77[T]	8	3,893	4.09	57.10	PC	coastal seawater around Geoje island	GCA_000622385.1	[14]
Sulfitobacter pontiacus 3SOLIMAR09	25	3,282	3.45	60.40	PC	water samples from Cala Figuera harbors	GCA_000647675.1	[15]
Sulfitobacter sabulilitoris HSMS-29[T]	19	3,711	3.95	64.40	PC	marine sand sample from the Yellow Sea	GCA_005887615.1	[16]
Sulfitobacter sediminilitoris JBTF-M27[T]	99	4,473	4.86	57.80	PC	tidal flat from Yellow Sea	GCA_010667575.1	[3]
Sulfitobacter undariae DSM 102234[T]	44	3,632	3.85	54.70	PC	Leachate from a brown algae reservoir	GCA_014196805.1	NA
Sulfitobacter faviae S5-53	7	4,696	3.86	62.30	PC	Scleractinia Coral *Favia veroni*	SRR6128330	[17]
Sulfitobacter faviae SCSIO W1865	5	3,939	4.03	62	PC	Cold water coral (115.3945°E，14.0321°N)	CP116423 - CP116427	This study
Sulfitobacter faviae SCSIO W1866	4	3,585	3.61	62	PC	Cold water coral (115°0.684′E，13°20.568′N)	CP116419-CP116422	This study

Table S2. List of lipid metabolism genes and antifreeze proteins in SCSIO W1865 and SCSIO W1866. Chr indicates chromosome, and none of the listed genes are identified in GIs and prophages.

		**SCSIO W1865**				**SCSIO W1866**			
**Lipid metabolism genes (**KEGG pathways**)**	Gene/Protein	location	start	stop	strand	location	start	stop	strand
ko00061 Fatty acid biosynthesis	*fabG*	Chr	1,587,104	1,587,841	+	Chr	1,862,587	1,863,324	-
		Plasmid1	812	1,600	+	Plasmid2	90,801	91,553	+
		Plasmid1	139,844	140,596	-				
		Plasmid1	241,292	242,122	-				
		.Plasmid3	7,047	7,817	+				
	fabI	Chr	1,096,001	1,096,756	-	Chr	1,740,020	1,740,826	+
		Chr	1,709,645	1,710,451	-	Chr	2,259,841	2,260,596	+
		Plasmid3	15,663	16,490	+				
	fabD	Chr	1,586,105	1,587,037	+	Chr	1,863,394	1,864,326	-
	fabB	Chr	1,096,802	1,098,031	-	Chr	2,258,566	2,259,795	+
	fabH	Chr	1,743,955	1,744,926	-	Chr	1,705,528	1,706,499	+
	fabA	Chr	1,100,302	1,100,811	-	Chr	2,255,785	2,256,294	+
	fadD	Chr	42,5961	42,7931	+	Chr	439,284	441,254	+
		Chr	714,733	716,553	+	Chr	2,599,562	2,601,382	-
	accC	Chr	1,565,187	1,566,539	-	Chr	1,883,867	18,85,219	+
	accA	Chr	3,047,359	3,048,321	+	Chr	2,954,054	2,955,016	+
	accD	Chr	3,115,733	3,116,692	-	Chr	3,022,341	3,023,300	-
	accB	Chr	1,566,549	1,567,058	-	Chr	1,883,351	1,883,857	+
	fabZ	Chr	2,045,423	2,045,893	-	Chr	1,362,828	1,363,298	+
	fabF	Chr	1,589,137	1,590,372	+	Chr	1,860,056	1,861,291	-
ko00071 Fatty acid degradation	*frmA*	Chr	1,188,250	1,189,365	+	Chr	2,161,938	2,163,053	-
	acd	Chr	1,175,271	1,176,545	-	Chr	1,913,344	1,914,495	-
		Chr	1,535,768	1,536,919	+	Chr	726,725	727,873	-
		Chr	2,676,061	2,677,209	+	Chr	2,174,764	2,176,038	+
		Plasmid1	19,472	20,629	-				
		Plasmid1	234,912	236,078	-				
	gcdH	Chr	2,662,718	2,663,941	-	Chr	739,991	741,214	+
	alkM	Chr	338,815	339,963	-	Chr	351,832	352,980	-

		Chr	2,362,841	2,363,839	+	Chr	1,031,046	1,032,047	-
	hcaD	Chr	309,077	310,282	+	Chr	322,099	323,304	+
	atoB	Chr	193,146	194,351	-	Chr	193,345	194,550	-
		Chr	377,223	378,356	+	Chr	390,180	391,313	+
		Chr	636,317	637,492	+	Chr	636,165	637,391	-
		Chr	2,677,998	2,679,212	+	Chr	724,722	725,936	-
		Chr	2,768,119	2,769,345	+	Chr	2,654,333	2,655,508	-
		Chr	3,023,892	3,024,998	+	Chr	2,930,960	2,932,066	+
		Chr	3,236,069	3,237,250	+	Chr	3,142,612	3,143,811	+
		Plasmid1	3,648	4,886	+	Plasmid2	85,823	86,995	-
		Plasmid1	15,468	16,619	-				
		Plasmid1	47,519	48,706	-				
		Plasmid1	144,405	145,574	+				
		Plasmid1	242,147	243,331	-				
		Plasmid4	46,536	47,735	-				
	paaF/echA	Chr	1,630,915	1,631,958	-	Chr	1,818,484	1,819,527	+
		Plasmid1	2,819	3,631	+				
	fadJ	Chr	2,679,233	2,681,434	+	Chr	722,500	724,701	-
	fadN	Chr	634,224	636,320	+	Chr	2,655,505	2,657,601	-
ko00120 Primary bile acid biosynthesis	*mcr*	Chr	170,891	171,994	+	Chr	171,086	172,189	+
		Plasmid1	13,440	14,576	-				
ko00121 Secondary bile acid biosynthesis	*hdhA*	Chr	2,994,623	2,995,423	-	Chr	2,901,688	2,902,488	-
ko00561 Glycerolipid metabolism	ALDH	Chr	399,417	401,780	-	Chr	412,740	415,103	-
	glpK	Plasmid2	174,082	175,578	-	Plasmid1	113,093	114,589	-
						Plasmid2	41,015	42,493	-
	lip	Chr	2,728,076	2,728,876	-	Chr	675,064	675,864	+
	plsX	Chr	1,744,923	1,745,999	-	Chr	1,704,455	1,705,531	+
	plsY	Chr	2,795,211	2,795,819	+	Chr	609,690	610,298	-
	gckA	Chr	1,086,720	1,088,018	+	Chr	2,268,602	2,269,873	-
	bgsB	Plasmid3	54,012	56,240	+				
ko00564 Glycerophospholipid metabolism	*gpsA*	Chr	111,046	111,999	-	Chr	111,045	111,998	-

	glpA	Plasmid2	181,666	183,255	-	Plasmid1	120,677	122,266	-
						Plasmid2	42,575	44,134	-
	pmtA	Chr	1,101,426	1,102,043	-	Chr	2,254,553	2,255,170	+
	cdsA	Chr	2,051,891	2,052,643	-	Chr	1,356,095	1,356,829	+
	pgsA	Chr	2,802,990	2803,655	-	Chr	601,854	602,519	+
	pcs	Chr	1,154,486	1,155,181	-	Chr	2,199,919	2,200,614	+
	pldB	Chr	1,270,742	1,271,689	-	Chr	2,079,894	2,080,841	+
	pgpA	Chr	2,004,262	2,004,753	+	Chr	1,403,770	1,404,261	-
	PLD1_2	Plasmid2	147,035	148,621	-	Plasmid1	86,048	87,634	-
	psd	Chr	1,102,835	1,103,527	-	Chr	2,253,069	2,253,761	+
	clsA_B	Chr	1,718,064	1,719,434	-	Chr	1,731,024	1,732,394	+
		Plasmid3	19,418	20,614	+				
	pssA	Chr	1,102,045	1,102,824	-	Chr	2,253,772	2,254,551	+
ko00600 Sphingolipid metabolism	*aslA*					Plasmid2	38501	40144	-
**antifreeze proteins**	Antifreeze protein	Chr	558,196	558,480	+	Chr	846,649	847,770	+
	Antifreeze protein	Chr	2,550,523	2,551,644	+	Chr	2,740,088	2,740,372	+

Table S3. List of secondary metabolites.

	Type	Start	Stop	Location	Most similar known Cluster	Similarity (%)
**SCSIO W1865**						
	Hserlactone	1,116,139	1,136,864	Chr.	-	-
	Terpene	1,715,834	1,736,598	Chr.	-	-
	β-lactone	2,220,504	2,247,181	Chr.	corynecin	13
	NRPS-PKS	2,399,621	2,451,645	Chr.	-	-
	Ectoine	3,219,386	3,229,778	Chr.	Ectoine	100
	β-lactone	261,811	287,790	Pla.1	-	-
**SCSIO W1866**						
	NRPS-PKS	954,610	1,006,634	Chr.	-	-
	β-lactone	1,149,913	1,176,591	Chr.	-	-
	Hserlactone	1,692,470	1,712,611	Chr.	-	-
	Hserlactone	2,218,302	2,239,027	Chr.	-	-
	Ectoine	3,125,995	3,136,387	Chr.	Ectoine	100

Table S4. List of sulfite oxidation genes in SCSIO W1865 and SCSIO W1866. The similarities showed the protein identities of enzymes in SCSIO W1866 with their homologues in SCSIO W1865.

	Gene	Start	Stop	Strand	Description	Similarity(%)
**SCSIO W1865**	*soxR*	1,552,644	1,552,306	-	Sulfur oxidation cycle transcriptional regulator	
	soxS	1,553,012	1,552,641	-	Periplasmic thiol--disulfide oxidoreductase	
	soxV	1,553,110	1,553,847	+	Cytochrome c-type biogenesis protein	
	soxW	1,553,876	1,554,460	+	Periplasmic thioredoxin	
	soxX	1,554,596	1,555,072	+	SoxY-Cys110-persulfide--sulfur compound transferase	
	soxY	1,555,193	1,555,612	+	Sulfur oxidation cycle carrier protein	
	soxZ	1,555,644	1,555,973	+	Sulfur oxidation cycle carrier protein,	
	soxA	1,556,018	1,556,869	+	SoxY-Cys110-persulfide--sulfur compound transferase	
	soxB	1,556,957	1,558,657	+	SoxZY-persulfide-sulfonate hydrolase,	
	soxC	1,558,731	1,559,993	+	Periplasmic sulfane dehydrogenase, molybdopterin-containing subunit	
	soxD	1,559,977	1,561,035	+	Periplasmic sulfane dehydrogenase, diheme c-type cytochrome subunit	
	soxG	1,905,756	1,906,688	+	Thiosulfate-induced periplasmic zinc metallohydrolase	
	soxH	1,906,685	1,907,632	+	Thiosulfate-induced periplasmic zinc metallohydrolase	
**SCSIO W1866**	*soxH*	1,540,873	1,539,926	-	Thiosulfate-induced periplasmic zinc metallohydrolase	97.46
	soxG	1,541,787	1,540,870	-	Thiosulfate-induced periplasmic zinc metallohydrolase	96.39
	soxD	1,890,395	1,889,337	-	Periplasmic sulfane dehydrogenase, diheme c-type cytochrome subunit	98.30
	soxC	1,891,641	1,890,379	-	Periplasmic sulfane dehydrogenase, molybdopterin-containing subunit	96.67
	soxB	1,893,415	1,891,715	-	SoxZY-persulfide-sulfonate hydrolase,	98.23
	soxA	1,894,354	1,893,503	-	SoxY-Cys110-persulfide--sulfur compound transferase	98.59
	soxZ	1,894,728	1,894,399	-	Sulfur oxidation cycle carrier protein	100
	soxY	1,895,179	1,894,760	-	Sulfur oxidation cycle carrier protein	100
	soxX	1,895,775	1,895,299	-	SoxY-Cys110-persulfide--sulfur compound transferase	100
	soxW	1,896,495	1,895,911	-	Periplasmic thioredoxin	96.90
	soxV	1,897,261	1,896,524	-	Cytochrome c-type biogenesis protein	99.18
	soxS	1,897,359	1,897,721	+	Periplasmic thiol--disulfide oxidoreductase	95.12
	soxR	1,897,718	1,898,056	+	Sulfur oxidation cycle transcriptional regulator	98.21

Table S5. List of DMSP related genes in SCSIO W1865 and SCSIO W1866. N and C indicate N terminus and C terminus of DmdA enzyme respectively. The similarities showed the protein identitiesof enzymes in SCSIO W1866 with their homologues in SCSIO W1865.

	Gene	Start	Stop	Location	strand	Similarity (%)
**SCSIO W1865**	*dmdA*	1,820,764	1,821,750	Chr.	+	
	dmdB1	1,955,426	1,953,798	Chr.	-	
	dmdB2	51,252	49,630	Plasmid 1	-	
	dmdC	2,673,993	2,675,774	Chr.	+	
	dmdD	3,240,580	3,241,356	Chr.	+	
	dddA	656,213	657,877	Chr.	+	
	dddC	1,635,853	1,634,354	Chr.	-	
	dddP	2,864,666	2,863,560	Chr.	-	
**SCSIO W1866**	*dmdA1*	1,629,782	1,629,081	Chr	-	99.04(N: 1-208)
	dmdA2	1,629,105	1,628,680	Chr	-	97.16(C: 227-367)
	dmdB	1,453,646	1,455,274	Chr	+	98.16
	dmdC	729,941	728,160	Chr	-	98.99
	dmdD	3,147,141	3,147,917	Chr	+	100.00
	dddA	3,093,407	3,091,794	Chr	-	56.50
	dddC	1,814,589	1,816,088	Chr	+	99.20
	dddP	3,117,358	3,116,249	Chr	-	30.98

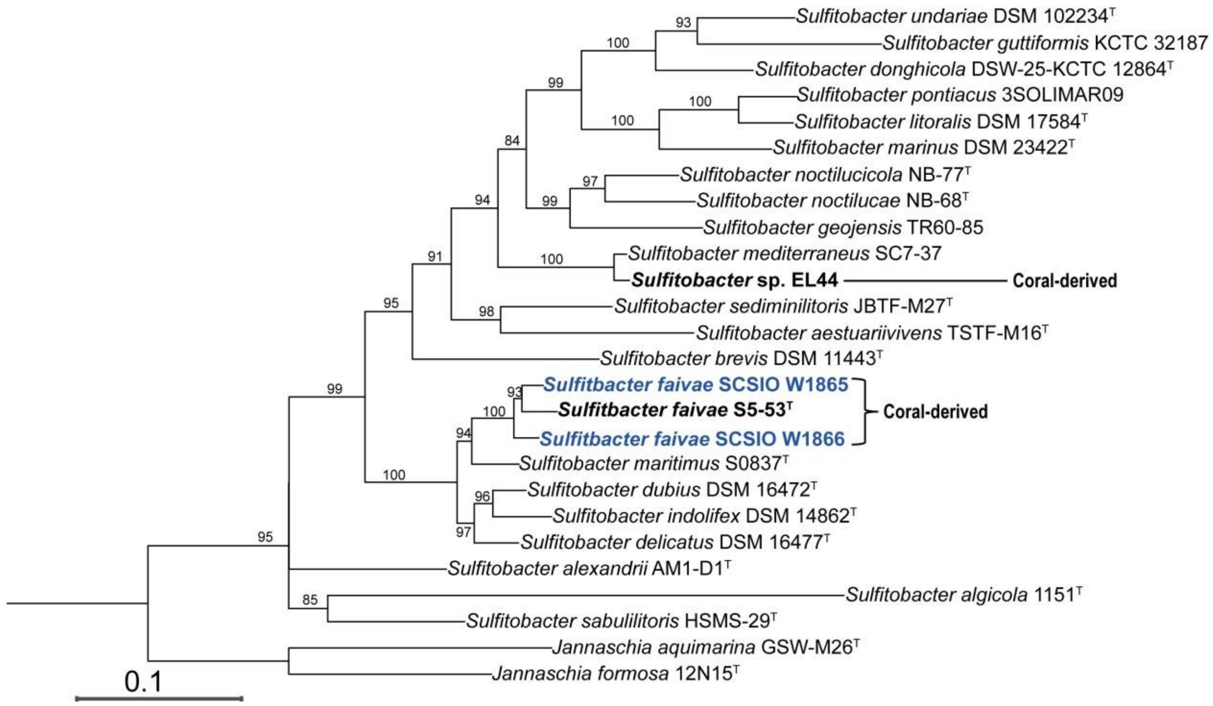

Figure S1. The maximum likelihood tree was constructed using PhyML 3.0 software [18] based on the genome sequences of SCSIO W1865, SCSIO W1866 and available *Sulfitobacter* genomes listed in Table S1. All genomes were aligned against the reference genome SCSIO W1865 using MUMmer (v3.0) [19] to generate whole-genome alignments and to identify single-nucleotide polymorphisms (SNPs) in the core genome, with repetitive regions removed. In total,10,636 SNPs were identified in these 26 genomes.Based on the concatenated SNPs, a maximum-likelihood tree with 1000 fast bootstrap replicates was inferred using PhyML 3.0 under GTR+I+G substitution model. Branch lengths are proportional to the number of nucleotide substitutions.

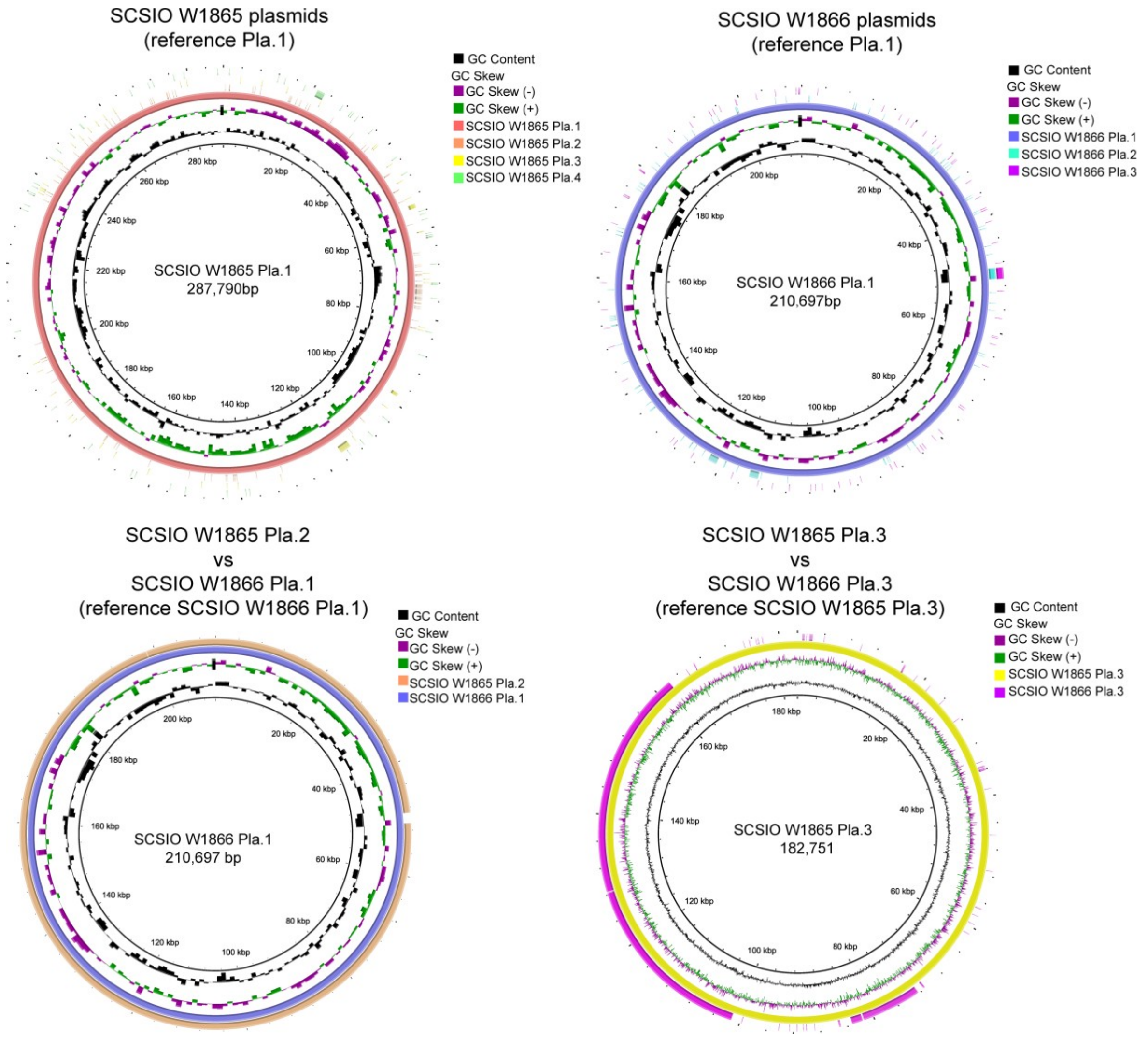

Figure S2. Comparison between different endogenous megaplasmids. The upper two panels showed the comparation among the endogenous megaplasmids in SCSIO W1865 and SCSIO W1866, separately. The lower panels showed the comparation between plasmids with identified similarities in nucleotide sequences.

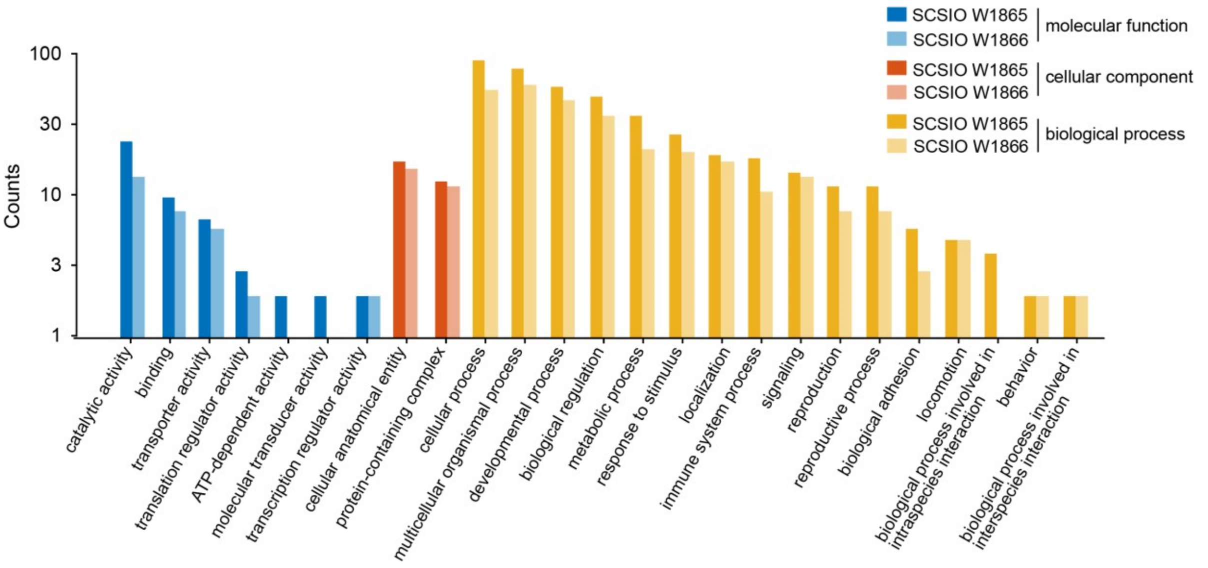

Figure S3. Geno Ontology classification of megaplasmids encoding genes. The genes encoded by megaplasmids in SCSIO W1866 and SCSIOW1865 were pooled separately. Then GO annotation was performed using eggnog [20], and GO level counts was performed using TBTOOLS software.

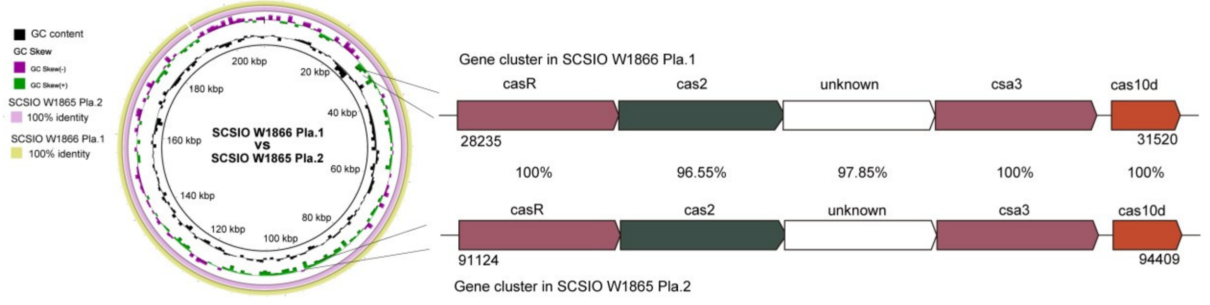

Figure S4. Loci and alignment of two incomplete CRISPR-Cas cluster. The genome sequence of SCSIO W1865 Pla.2 and SCSIO W1866 Pla.1 were aligned using BRIG. Their DNA sequences show high similarities (95.78%). The proteins in the predicted clusters were compared in NCBI and their identities were labeled. The number below SCSIO W1866 Pla.1 and SCSIO W1865 Pla.2 showed the start of the first gene and the stop of the last gene, respectively.

References

1. Voget, S.; Diaz Valerio, S. M.; von Hoyningen-Huene, A. J.; Nattramilarasu, P. K.; Vollheyde, K.; Xiao, S.; Daniel, R., Genome sequence of *Jannaschia aquimarina* GSW-M26, a member of the *Roseobacter* clade. *Genome Announc* **2015,** 3, (2), e00353-15.
2. Zhang, R.; Wang, C.; Wang, X. T.; Mu, D. S.; Du, Z. J., *Jannaschia formosa* sp. nov., isolated from marine saltern sediment. *Int. J. Syst. Evol. Microbiol.* **2019,** 69, (7), 2037-2042.
3. Park, S.; Chen, S.; Lee, J. S.; Kim, W.; Yoon, J. H. Description of *Sulfitobacter sediminilitoris* sp. nov., isolated from a tidal flat. *FEMS Microbiol. Lett.* **2020,** 367, fnaa074.
4. Yang, Q.; Ge, Y. M.; Iqbal, N. M.; Yang, X.; Zhang, X. L., *Sulfitobacter alexandrii* sp. nov., a new microalgae growth-promoting bacterium with exopolysaccharides bioflocculanting potential isolated from marine phycosphere. *Antonie van Leeuwenhoek* **2021,** 114, (7), 1091-1106.
5. Wang, C. N.; Liu, Y.; Wang, J.; Du, Z. J.; Wang, M. Y., *Sulfitobacter algicola* sp. nov., isolated from green algae. *Arch. Microbiol.* **2021,** 203, (5), 2351-2356.
6. Labrenz, M.; Tindall, B. J.; Lawson, P. A.; Collins, M. D.; Schumann, P.; Hirsch, P., *Staleya guttiformis* gen. nov., sp. nov. and *Sulfitobacter brevis* sp. nov., alpha-3-Proteobacteria from hypersaline, heliothermal and meromictic antarctic Ekho Lake. *Int. J. Syst. Evol. Microbiol.* **2000,** 50 Pt 1, 303-313.
7. Ivanova, E. P.; Gorshkova, N. M.; Sawabe, T.; Zhukova, N. V.; Hayashi, K.; Kurilenko, V. V.; Alexeeva, Y.; Buljan, V.; Nicolau, D. V.; Mikhailov, V. V.; Christen, R., *Sulfitobacter delicatus* sp. nov. and *Sulfitobacter dubius* sp. nov., respectively from a starfish (*Stellaster equestris*) and sea grass (*Zostera marina*). *Int. J. Syst. Evol. Microbiol.* **2004,** 54, (Pt 2), 475-480.
8. Bartling, P.; Vollmers, J.; Petersen, J., The first world swimming championships of *roseobacters*-Phylogenomic insights into an exceptional motility phenotype. *Syst. Appl. Microbiol.* **2018,** 41, (6), 544-554.
9. Park, J. R.; Bae, J. W.; Nam, Y. D.; Chang, H. W.; Kwon, H. Y.; Quan, Z. X.; Park, Y. H., *Sulfitobacter litoralis* sp. nov., a marine bacterium isolated from the East Sea, Korea. *Int. J. Syst. Evol. Microbiol.* **2007,** 57, (Pt 4), 692-695.
10. Lian, F. B.; Li, Y. Q.; Zhang, J.; Jiang, S.; Du, Z. J., *Sulfitobacter maritimus* sp. nov., isolated from coastal sediment. *Int. J. Syst. Evol. Microbiol.* **2021,** 71, (2).
11. Raimundo, I.; Silva, S. G.; Costa, R.; Keller-Costa, T., Bioactive secondary metabolites from octocoral-associated microbes-new chances for blue growth. *Mar Drugs* **2018,** 16, (12), 485.
12. Sweet, M.; Villela, H.; Keller-Costa, T.; Costa, R.; Romano, S.; Bourne, D. G.; Cardenas, A.; Huggett, M. J.; Kerwin, A. H.; Kuek, F.; Medina, M.; Meyer, J. L.; Muller, M.; Pollock, F. J.; Rappe, M. S.; Sere, M.; Sharp, K. H.; Voolstra, C. R.; Zaccardi, N.; Ziegler, M.; Peixoto, R., Insights into the cultured bacterial fraction of corals. *mSystems* **2021,** 6, (3), e01249-20.
13. Almeida, J. F.; Marques, M.; Oliveira, V.; Egas, C.; Mil-Homens, D.; Viana, R.; Cleary, D. F. R.; Huang, Y. M.; Fialho, A. M.; Teixeira, M. C.; Gomes, N. C. M.; Costa, R.; Keller-Costa, T., Marine sponge and octocoral-associated bacteria show versatile secondary metabolite biosynthesis potential and antimicrobial activities against human pathogens. *Mar. Drugs* **2023,** 21, (1), 34.
14. Kwak, M. J.; Lee, J. S.; Lee, K. C.; Kim, K. K.; Eom, M. K.; Kim, B. K.; Kim, J. F., *Sulfitobacter geojensis* sp. nov., *Sulfitobacter noctilucae* sp. nov., and *Sulfitobacter noctilucicola* sp. nov., isolated from coastal seawater. *Int. J. Syst. Evol. Microbiol.* **2014,** 64, (Pt 11), 3760-3767.
15. Mas-Llado, M.; Pina-Villalonga, J. M.; Brunet-Galmes, I.; Nogales, B.; Bosch, R., Draft genome sequences of two isolates of the *Roseobacter* group, *Sulfitobacter* sp. strains 3SOLIMAR09 and 1FIGIMAR09, from harbors of Mallorca Island (Mediterranean Sea). *Genome Announc* **2014,** 2, (3), e00350-14.
16. Park, S.; Kim, I. K.; Lee, J. S.; Yoon, J. H., *Sulfitobacter sabulilitoris* sp. nov., isolated from marine sand. *Int. J. Syst. Evol. Microbiol.* **2019,** 69, (10), 3230-3236.
17. Kumari, P.; Bhattacharjee, S.; Poddar, A.; Das, S. K., *Sulfitobacter faviae* sp. nov., isolated from the coral *Favia veroni*. *Int. J. Syst. Evol. Microbiol.* **2016,** 66, (10), 3786-3792.
18. Guindon, S.; Dufayard, J. F.; Lefort, V.; Anisimova, M.; Hordijk, W.; Gascuel, O., New algorithms and methods to estimate maximum-likelihood phylogenies: assessing the performance of PhyML 3.0. *Syst Biol* **2010,** 59, (3), 307-21.
19. Kurtz, S.; Phillippy, A.; Delcher, A. L.; Smoot, M.; Shumway, M.; Antonescu, C.; Salzberg, S. L., Versatile and open software for comparing large genomes. *Genome Biol.* **2004,** 5, (2), R12.
20. Cantalapiedra, C. P.; Hernandez-Plaza, A.; Letunic, I.; Bork, P.; Huerta-Cepas, J., eggNOG-mapper v2: functional annotation, orthology assignments, and domain prediction at the metagenomic scale. *Mol. Biol. Evol.* **2021,** 38, (12), 5825-5829.
